# Simplified Deep Reinforcement Learning Approach for Channel Prediction in Power Domain NOMA System

**DOI:** 10.3390/s23219010

**Published:** 2023-11-06

**Authors:** Mohamed Gaballa, Maysam Abbod

**Affiliations:** Department of Electronic and Electrical Engineering, Brunel University London, Uxbridge UB8 3PH, UK; maysam.abbod@brunel.ac.uk

**Keywords:** DRL, DQN, Q-learning, LSTM, NOMA

## Abstract

In this work, the impact of implementing Deep Reinforcement Learning (DRL) in predicting the channel parameters for user devices in a Power Domain Non-Orthogonal Multiple Access system (PD-NOMA) is investigated. In the channel prediction process, DRL based on deep Q networks (DQN) algorithm will be developed and incorporated into the NOMA system so that this developed DQN model can be employed to estimate the channel coefficients for each user device in NOMA system. The developed DQN scheme will be structured as a simplified approach to efficiently predict the channel parameters for each user in order to maximize the downlink sum rates for all users in the system. In order to approximate the channel parameters for each user device, this proposed DQN approach is first initialized using random channel statistics, and then the proposed DQN model will be dynamically updated based on the interaction with the environment. The predicted channel parameters will be utilized at the receiver side to recover the desired data. Furthermore, this work inspects how the channel estimation process based on the simplified DQN algorithm and the power allocation policy, can both be integrated for the purpose of multiuser detection in the examined NOMA system. Simulation results, based on several performance metrics, have demonstrated that the proposed simplified DQN algorithm can be a competitive algorithm for channel parameters estimation when compared to different benchmark schemes for channel estimation processes such as deep neural network (DNN) based long-short term memory (LSTM), RL based Q algorithm, and channel estimation scheme based on minimum mean square error (MMSE) procedure.

## 1. Introduction

It can be noticed that the high energy consumption by the connected terminals in the current wireless networks can create an essential challenge in designing the upcoming 6G wireless systems [1]. Therefore, it is important to consider this energy consumption issue in future wireless communication networks, and at the same time, we need to maintain the required quality of service (QoS) for devices or services in that networks. Basically, NOMA system utilizes a superposition coding (SC) procedure that involves multiplexing different signals related to different users before transmission, which can contribute to the energy efficient transmission scheme. Moreover, NOMA system can also be designated to ensure the desired quality of service (QoS) levels for all superimposed user devices. Numerous research efforts have been dedicated to NOMA system in order to find an efficient strategy for different challenging tasks such as power allocation, beamforming, and channel assignment [2].

Recently, many authors have investigated different machine learning algorithms and artificial intelligence tools to optimize the resource allocation problems in NOMA system [3]. Furthermore, reinforcement learning (RL) based Q-learning algorithm and deep reinforcement learning based Q network (DQN) have gained a remarkable interest among authors in various fields. The Q-learning algorithm is a subclass of reinforcement learning that depends on Q-tables to store the optimal Q-values for each state-action pair in order to maximize the future reward in the system. Alternatively, deep reinforcement learning-based Q network (DQN) algorithm is mainly dependent on adopting hidden layers that can effectively enhance network convergence and system performance.

### 1.1. Related Works

In the context of optimizing communication systems, several works have employed the Q-learning algorithm to enhance the performance of wireless networks based on different perspectives. The work in [4] applied the Q-learning algorithm to introduce a framework for enabling mobile edge computing in NOMA system. In [5], authors suggested a dynamic reinforcement learning scheme for power allocation in order to jointly maximize the sum rate and the spectral efficiency in MIMO-NOMA system when smart jamming is considered. The authors applied the Q-learning algorithm to allocate a certain power level to each user terminal, to mitigate the jamming effects. 

Basically, by incorporating deep learning into RL, deep reinforcement learning (DRL) can address the challenges associated with Q-learning in terms of Q-table storage. Based on that, the work in [6] introduced a deep Q-network (DQN) to model a multiuser NOMA offloading problem, while the work in [7], proposed a power allocation technique based on deep reinforcement learning in cache-assisted NOMA system. Furthermore, authors in [8] introduced a DRL based actor-critic algorithm to handle the dynamic power allocation policy. Likewise, DRL based actor-critic algorithm was also applied in [9] to attain the optimal policy for user scheduling and resource allocation in HetNets. In [9], the authors designed the actor network in order to decide the policy that can select a stochastic action based on Gaussian distribution, while the critic network role is to evaluate the value function and guides the actor network to discover or learn the optimal policy. 

Deep reinforcement learning was also introduced in [10] to arrive at a sub-optimal power allocation scheme for an uplink multicarrier NOMA cell. The work in [11], considered a joint channel assignment and power distribution procedure in NOMA system. Authors in [11], derived a near-optimal power allocation scheme by considering two users per channel, and the channel assignment was performed using deep reinforcement learning algorithm to boost the overall sum rate while the minimum rate for each user device is considered. 

### 1.2. Research Gap and Significance

Several machine learning (ML) algorithms have been suggested to clearly address diverse issues in wireless networks such as channel assignment, beamforming, and power allocation. Also, several RL algorithms have been proposed to handle the channel estimation task in wireless communication systems. However, most of the current research that covers the channel prediction task in the NOMA system is mainly dependent on deep neural networks (DNN) which include some sort of complexity in the network structure. Hence, in this work, we aim to introduce a deep reinforcement learning scheme based on a simplified DQN approach to reduce the complexity structure and at the same time enhance the channel estimation process. Furthermore, to the best of the authors’ knowledge, there is no study that explores the utilization of deep reinforcement learning (DRL) based deep Q network (DQN) algorithm for estimating the channel parameters for user devices in the NOMA system. In addition, and to the best of the authors’ knowledge, there is no study that has investigated the performance of NOMA system when both the DQN algorithm that used as channel estimator and the optimized power scheme are jointly implemented for user detection in NOMA system. 

It is worth mentioning that unlike classical deep learning algorithms, which mainly depend on learning from a training data set, the proposed DQN algorithm is developed based on the LSTM network to adapt to the variations in the channel and to dynamically enhance the system performance based on the interaction with the environment. 

### 1.3. Contributions to Knowledge

In this work, the contributions can be summed up as shown:

- A simplified DQN structure is proposed to demonstrate how RL based DQN algorithm is developed to predict the channel parameters for each user in the NOMA cell in Rayleigh fading channels.
- Investigate the combination between the RL algorithm and the LSTM model, to compose the simplified DQN structure in order to be utilized as a channel estimator.
- Validate the efficiency of the proposed DQN scheme, by establishing different benchmark schemes for comparison. Three different simulation environments are established as follows: (1) Channel prediction scheme based on standard minimum mean square error (MMSE) procedure [12]; (2) Standard DNN based on LSTM network for channel prediction applied in [13], (3) The RL based Q-algorithm for channel prediction applied in [14]. The simulation outcomes of these benchmark schemes were compared with the results of our proposed DQN model, and the results emphasized that reliability can be guaranteed by our developed DQN algorithm for predicting channel parameters even when the number of users in NOMA cell is increased.
- Simulate the impact of integrating the simplified DQN structure for channel prediction and the optimized power scheme derived in [13] for the purpose of multiuser detection in the power domain NOMA system.

The remainder of this paper is structured as follows. Section 2 describes the system model. The Deep Reinforcement Learning Framework is presented in Section 3. The Channel Estimation Based DQN Algorithm is discussed in Section 4. DQN Operation and framework are discussed in Section 5. DQN Dataset Generation is introduced in Section 6. Section 7 discusses the DQN Policy and Algorithm. DQN state space, action space, and reward are introduced in Section 8. Detailed DQN Procedure and workflow are listed in Section 9. Complexity analysis is also discussed in Section 10. The simulation environment is described in Section 11, and simulation results are presented in Section 12. Finally, conclusions are given in Section 13.

## 2. System Model

In a NOMA cell, numerous user devices can be served via the same resource block (RB) by employing the power domain (PD) in both uplink and downlink transmissions. In this paper, we are considering a downlink NOMA cell, where the BS can serve distinct types of users or devices at the same time via different fading channels. At the transmitter side, the BS can assign a specific channel or subcarrier to every set of user devices, and the signals of these devices can be multiplexed using unique power levels. At the receiver side, each user device will receive the desired signal beside the undesirable signals related to other devices in the same channel that will be considered either as interference or noise. The undesirable received signals will be considered as noise if the power level of the desired signal is high, otherwise, these additional signals will be regarded as interference. To decode the desired signal, each user device will use the successive interference cancelation (SIC) procedure. The SIC technique will first decode the signal with the highest power level and then subtract that signal from the principal signal, and this process will continue until the desired signal is decoded.

Typically, before applying the SIC procedure at the receiver side, the channel parameters for each user need to be available or estimated to perform the equalization process. Also, to calculate the data rate or channel capacity for each user, we need to calculate the signal to interference plus noise ratio (SINR), and SINR itself includes the channel gain ${\left|h_{i}\right|}^{2}$, where $h_{i}$ represents the fading channel between the BS and user device *i*. In the NOMA scenario, the data rate $R_{i}$ for user device *i* can be expressed as follows:

(1) $$R_{i}=\log_{2}\left(1+\frac{P_{T}\alpha_{i}\eta_{i}}{\sum_{j=1}^{i-1}P_{T}\alpha_{j}\eta_{i}+1}\right)\;$$

where $\alpha_{i}$ is the power allocation factor for user device *i*, and $\eta_{i}$ is the channel to noise ratio (CNR) for user i and $P_{T}$ is the total power assigned by the BS. The channel to noise ratio $\eta_{i}$ for user i, can be expressed as follows:

(2) $$\eta_{i}=\frac{{\left|h_{i}\right|}^{2}}{\sigma_{n}^{2}}\;$$

where ${\left|h_{i}\right|}^{2}$ is the channel gain for user device *i*, and $\sigma_{n}^{2}$ is the noise power. In this work, we are considering a downlink NOMA system, and the total number of devices in the cell is *N*. In the NOMA cell, all signals related to the *N* devices are combined, and the BS will transmit this composed signal to all users in the cell. The composed signal X can be represented as follows [15]:

(3) $$X=\overset{N}{\underset{i=1}{\sum }}\sqrt{P_{T}\alpha_{i}}x_{i}\;i=1,2,\ldots .,N\;$$

where $x_{i}$ is the desired signal for user device *i*. The composed transmitted signal X can be received at the receiver side of each user terminal, with path loss and Additive White Gaussian noise (AWGN), hence the received signal Y can be represented as

(4) $$Y=\overset{N}{\underset{i=1}{\sum }}\sqrt{P_{T}\alpha_{i}}h_{i}x_{i}+n\;i=1,2,\ldots .,N\;$$

where $h_{i}$ is the fading channel between BS and user device *i* and n denotes the AWGN component. After receiving the composed signal and estimating the channel parameters, the receiver at each user device will activate the SIC procedure to decode the desired signal. In PD-NOMA, distinct power levels will be given to user terminals in the cell, and the highest power level will be given to the user device with the lowest CNR, while the lowest power level will be given to the user device with the highest CNR. Therefore, if user devices have the following CNRs:

(5) $$\eta_{1}>\eta_{2}>.\;.\;.\;.>\eta_{N}$$

Then, these user devices will be assigned power levels as follows:

(6) $$P_{1}<P_{2}<.\;.\;.\;.<P_{N}\;$$

The SINR for user device *i* can be represented as shown:

(7) $$SINR_{i}=\frac{P_{T}\alpha_{i}\eta_{i}}{\sum_{j=1}^{i-1}P_{T}\alpha_{j}\eta_{i}+1}\;i=1,2,\ldots .,N$$

The BS can allocate power $P_{i}$ to any user terminal as shown in the following expression [15]:

(8) $$P_{i}=\left(P_{T}-\left(\overset{i-1}{\underset{j=1}{\sum }}P_{T}\alpha_{j}\right)\right)\geq P_{th}\;$$

The expression in (8), can be interpreted as follows: for proper achievement for the SIC process, the user device with low CNR must have a higher power level than the sum of power levels for other devices that have high CNR.

Based on the aforementioned analysis, in what follows we will consider the scenario for three users downlink PD-NOMA system, and we will provide some sort of mathematical analysis for the achievable capacity for each user when both perfect SIC and imperfect SIC are applied [16]. As indicated before, BS can send the superposition coded signal X which can be expressed as

(9) $$\;X=\sqrt{P_{t}}\left(\sqrt{\alpha_{n}}x_{n}+\sqrt{\alpha_{m}}x_{m}+\sqrt{\alpha_{f}}x_{f}\right)\;$$

where $\alpha_{n}$, $\alpha_{m}$, and $\alpha_{f}$ are the power factors allocated to the near user, middle user, and far user, respectively. Likewise, $x_{n}$, $x_{m}$, and $x_{f}$ denote the desired symbols related to the near, middle, and far users respectively. Hence, the signal received at far user can be represented as follows:

(10) $$\;\;\;y_{f}=Xh_{f}+n_{f}$$

where $h_{f}$ represent the fading channel among BS and the far user, while $n_{f}$ represents the AWGN noise component at far user with zero mean and $\sigma^{2}$ variance. The received signal at far user can be expressed in details as follows:

(11) $$y_{f}=\sqrt{P_{t}\alpha_{f}}x_{f}h_{f}+\sqrt{P_{t}}\left(\sqrt{\alpha_{m}}x_{m}+\sqrt{\alpha_{n}}x_{n}\right)h_{f}+n_{f}$$

The 1st term in (11) represents the desired signal for far user, but the 2nd term denotes the interference term from the middle and near users. Far user is usually described by poor channel condition and his particular signal $x_{f}$ can be assigned additional power by BS compared to other users. Thus, according to the SIC scheme, far user can directly decode his own signal $x_{f}$ from received signal $y_{f}$. The possible rate for far user $R_{f}$ could be expressed as follows:

(12) $$R_{f}=\log_{2}\left(1+\frac{\eta_{f\;}P_{t}\alpha_{f}}{\eta_{f\;}P_{t}\left(\alpha_{n}+\alpha_{m}\right)+1}\right)\;$$

Likewise, the attainable bit rate for the middle user $R_{m}$ in the case of perfect SIC, can be expressed as follows:

(13) $$R_{m}=\log_{2}\left(1+\frac{\eta_{m\;}P_{t}\alpha_{m}}{\eta_{m\;}P_{t}\left(\alpha_{n}\right)+1}\right)\;$$

Typically, the user near the BS has a good channel condition; therefore, his signal $x_{n}$ is usually assigned low power level. Therefore, at near user side when perfect SIC is applied, firstly immediate decoding for far user signal $x_{f}$ is accomplished, then it is removed from the composite signal. Next, the middle user signal $x_{m}$ is decoded and removed from the remaining signal. Finally, the near user achieved rate $R_{n}$ can be expressed as follows:

(14) $$R_{n}=\log_{2}\left(1+\eta_{n\;}P_{t}\alpha_{n}\right)\;\;$$

In the case of imperfect SIC, the attainable bit rate for the middle user can be expressed as:

(15) $$R_{m}=\log_{2}\left(1+\frac{\eta_{m\;}P_{t}\alpha_{m}}{\epsilon \;\eta_{m\;}P_{t}\left(\alpha_{f}\right)+\eta_{m\;}P_{t}\left(\alpha_{n}\right)+1}\right)\;\;$$

where $\epsilon \;\eta_{m\;}P_{t}\left(\alpha_{f}\right)$ represents the error residual term from far user signal decoding. Likewise, the attainable bit rate for the near user in case of imperfect SIC can be expressed as:

(16) $$\;R_{n}=\log_{2}\left(1+\frac{\eta_{n\;}P_{t}\alpha_{n}}{\epsilon \;\eta_{n\;}P_{t}\left(\alpha_{f}\right)+\epsilon \;\eta_{n\;}P_{t}\left(\alpha_{m}\right)+1}\right)\;$$

where $\epsilon \;\eta_{n\;}P_{t}\left(\alpha_{f}\right)$ is the error residual term from far user signal decoding and $\epsilon \;\eta_{n\;}P_{t}\left(\alpha_{m}\right)$ is the error residual term from middle user signal decoding.

## 3. Deep Reinforcement Learning Framework

In this section, we will introduce the concept of deep reinforcement learning (DRL), which is a special case of reinforcement learning procedure [17,18]. Reinforcement learning is a fork of machine learning, where an agent interacts with the environment to carry out the best sequences of actions that can maximize the expected future reward in an interactive environment. Generally, reinforcement learning can be classified as single-agent or multi-agent based on the quantity of agents in the environment. In the scenario of a single agent RL, the agent needs to recognize the entire states in the environment and the decision-making task can be modeled as a Markov decision process (MDP) framework. In this work, our proposed DQN structure assumes a single agent, and the best sequence of actions that can be chosen by the agent will be generated based on the adopted deep neural network (DNN). 

The fundamental elements in the deep reinforcement learning (DRL) algorithm can be listed as follows [14,18]:

1. **Observations**: the continuous measurements of the properties of the environment, and all of the observed properties in the environment can be included in the state space S.
2. **States**: the discret observation at time step *t* can be denoted as state $s_{t}\in S$.
3. **Actions**: an action $a_{t}$ is one of the valid decisions that the agent can select at time step *t* from the action space A.
4. **Policy**: a policy denoted by π(.), is the criteria that control how to select a certain action at any given state while interacting with the environment.
5. **Rewards**: the immediate reward $r_{t}$, is obtained after an agent carries out a specific action $a_{t}$ in a given state $s_{t}$, which leads to moving to a new state $s_{t+1}$.
6. **State-action value function**: denoted by $Q_{\pi }\left(s,a\right)$, and represents the expected discounted reward when the agent starts at a certain state $s_{t}$ and selects a specific action $a_{t}$ based on the policy *π*.

In the DQN framework, when an agent selects an action $a_{t}$ at a given time step *t*, the agent’s state will change from the current state $s_{t}$ to the subsequent state $s_{t+1}$ and as a result of this transition, the agent will receive an immediate reward $r_{t}$ from the environment. Based on that scenario, the network can generate an experience tuple $e=\left(s_{t},\;a_{t},\;r_{t},\;s_{t+1}\right)$ that can be stored in the experience replay buffer D. The primary target of the agent in RL scheme is to maximize the long-term cumulative discounted reward $R_{t}^{\gamma }$, which can be defined as follows [14,18]:

(17) $$\;R_{t}^{\gamma }=\overset{\infty }{\underset{i=0}{\sum }}\gamma^{i}r_{t+i}\;$$

where γ is the discount factor. To enhance the $R_{t}^{\gamma }$, an optimal policy $\pi^{*}$ is essential to map the best actions to states. In other words, the optimal policy $\pi^{*}$ can significantly assist the agent in deciding which action should be selected at any given state, to satisfy the optimal long-term cumulative reward. Typically, the state action Q-value function is defined as the expectation of the cumulative discounted reward $R_{t}^{\gamma }$. Overall, we can notice that based on the current state $s_{t}$, the considered policy π, and the selected action $a_{t}$, the state-action Q value function can be further expressed as follows [14,19]:

(18) $$Q_{\pi }\left(s_{t},a_{t}\right)=E\left[R_{t}^{\gamma }\left|s_{t}\right.,a_{t}\right]=E\left[\overset{\infty }{\underset{i=0}{\sum }}\gamma^{i}r_{t+i}\left|s_{t}\right.,a_{t}\right]\;=E\left[r_{t}+\gamma Q_{\pi }\left(s_{t+1},a_{t+1}\right)\left|s_{t}\right.,a_{t}\right]\;$$

where $E\left[\;.\right]$ denotes the expectation parameter. When the optimal policy $\pi^{*}$ is applied for maximizing all states and action pairs, then the optimal Q-value function $Q_{\pi^{*}}\left(s_{t},a_{t}\right)$ that follows the optimal policy $\pi^{*}$ can be expressed as follows:

(19) $$Q_{\pi^{*}}\left(s_{t},a_{t}\right)=E\left[r_{t}+\gamma Q_{\pi^{*}}\left(s_{t+1},a_{t+1}\right)\left|s_{t}\right.,a_{t}\right]\;$$

The expression in (19) is known as the Bellman equation. The benefit of the Bellman equation is to represent the state-action Q-value function into two components: the instantaneous reward $r_{t}$ and the long-term discounted reward. However, the Bellman equation is nonlinear, and hence, there is no closed form solution to it. As a result, an iterative procedure such as the Q-learning algorithm has emerged to converge the Bellman equation to obtain the optimal Q-value function [18,19]. On the other hand, the computation of the Q-learning algorithm may become more complex in multi-user environments that have huge state and action spaces, and as a result, the size of the Q-table will be extremely large. Hence, the regular solution to this limitation is to estimate the Q-values using a function approximator, by adopting hidden layers, which is the core component in our developed deep Q network.

**Figure 1:**
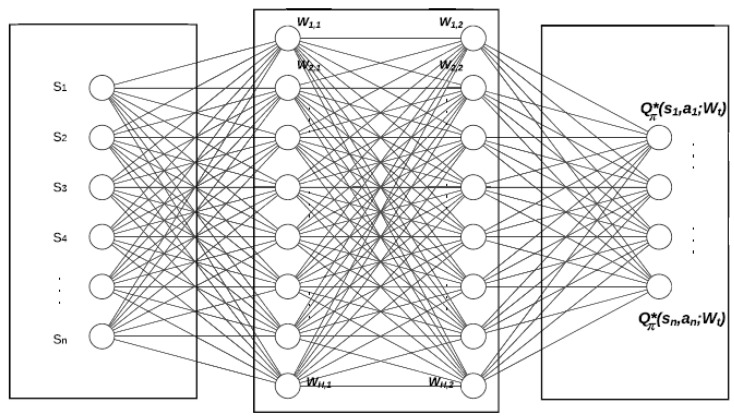
DQN basic structure with two hidden layers.

The basic DQN architecture is shown in Figure 1, and it consists of three main phases: The first phase represents the input layer that includes the current states of the environment. The second stage includes the hidden layers that act as a function approximator. Mainly in the hidden layers, the Rectified Linear Unit (ReLU) activation function is applied to compute the hidden layer values. The primary gain of utilizing ReLU as an activation function is the computational efficiency [20], which may lead to faster convergence. At the end phase, the output layer is responsible for predicting the optimal state-action value function, $Q_{\pi^{*}}\left(s,a,W_{t}\right)$, where $W_{t}$ is the updated weights of the hidden layers at time instant *t*.

## 4. Channel Estimation Based DQN Algorithm

**Figure 2:**
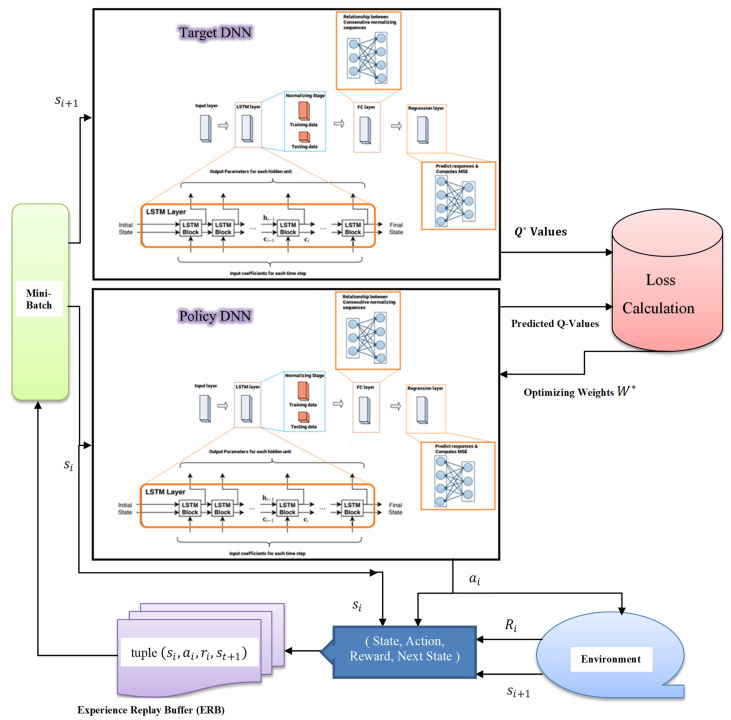
Proposed DQN Architecture.

In this section, the simplified DRL structure will be introduced, and Figure 2 illustrates the architecture of the simplified DRL scheme that mainly relies on the DQN algorithm and LSTM network to achieve the most appropriate performance. The DQN network will be trained, and the weights of the hidden layers will be updated to approximate the state-action value function $Q_{\pi }\left(s,a\right)$. As indicated in the aforementioned discussion, each experience tuple is described as $e_{t}=\left(s_{t},a_{t},r_{t},s_{t+1}\right)$, and all experience tuples will be stored in an experience replay buffer $D=\left\{e_{1}\;e_{2}\;e_{3}\;\ldots \;e_{t}\right\}$, and these experience tuples can be utilized to train the DQN using the gradient descent algorithm [21]. It is optimum for the DQN algorithm to exploit all available experience tuples in each training iteration, but this will be costly when the training set is huge. A more effective procedure is to update the DQN weights in each iteration using an arbitrary subset from the replay buffer D, and this subset is described as a mini batch. Based on the architecture of the proposed DQN structure shown in Figure 2, it can be noticed that the loss function can be computed based on the difference between the output of the target DNN and the output of the policy DNN. Hence, the loss function can be defined as follows [18,19]:

(20) $$L\left(W\right)=\underset{e\in D}{\sum }{\left(r_{t}+\gamma \;maxQ_{\pi^{*}}\left(s_{t+1},a_{t+1},\hat{W}\right)-Q_{\pi^{*}}\left(s_{t},a_{t},W\right)\right)}^{2}\;$$

where $L\left(W\right)$ denotes the DQN loss function for a random mini batch sampled from the replay buffer D at time slot *t* and $\hat{W}$ represents the nearly static weights in the target DNN and these weights are mainly updated every *T* time steps. To minimize the loss function $L\left(W\right)$, the weights W of the policy DNN will be updated every *t* time step using a stochastic gradient descent (SGD) algorithm applied on a batch of random samples selected from the replay buffer D. Typically, the SGD algorithm can update the weights of the policy DNN W in an iterative process with a learning rate of μ > 0 as follows [21]:

(21) $$\;W_{t+1}=W_{t}-\mu \;\nabla L_{t}\left(W_{t}\right)\;$$

## 5. Proposed DQN Operation and Phases

**Phase 1:** Initialization and generation of training data

1. Perform a few random actions with the environment to initialize the experience replay data.
2. Initialize the weights for the policy DNN and copy these weights to the Target DNN.
3. Starting with the first time step,
  - Based on the initial interaction with the environment, random states can be generated to be used as input for the policy DNN.
  - The policy DNN will predict the Q-values for all actions that can be decided in the current state, and then those Q-values will be inspected to select or identify a certain Q-value based on the most suitable action.
  - Based on the selected and executed action, the experience replay will receive the reward and move to the next state.
  - The experience replay will store the results in the replay buffer.
  - Each result will be considered as a sample training data, that can be later used as training data.

**Phase 2:** Select a random batch for training

1. Select a batch of random samples from the replay buffer and use these samples as an inputs for both the policy DNN and the target DNN.
2. From the random sample, use the current state as input to the policy DNN.
3. The policy DNN can predict the Q-values for all actions that can be performed in the current state.
4. Based on the decided or selected action, the policy DNN will identify the predicted Q-value.
5. The next state from the selected random sample will be used as input to the Target DNN.
6. The Target network will predict the Q-values for all actions that can be performed in the next state, then the Target DNN will select the maximum of those Q-values.

**Phase 3:** Get the Target Q-value

1. The Target Q-value can be decided based on two components
  - The immediate reward from the environment
  - The max Q value that has been predicted by the target DNN in the next state

**Phase 4:** Compute the Loss function

1. Compute the loss function between the Target Q value and the predicted Q Value in terms of mean squared error (MSE).

**Phase 5:** Back-propagate the Loss function

1. Back-propagate the loss in order to update the weights of the policy DNN using SGD.
2. At this stage, the weights of the Target DNN are not updated and remain fixed, and this completes the processing for this time step.

**Phase 6:** Repeat for next time step

1. The process will be repeated for the next time step.
  - The policy DNN weights have been updated but not the Target DNN.
  - This allows the policy DNN to learn to predict more accurate Q-values, while the weights for the target DNN remain fixed for a while.
2. After *T* time steps, copy the policy DNN weights to the Target DNN. This step will enable the Target DNN get the updated weights so that it can also predict more accurate target Q-values.

Long-short term memory (LSTM) network is a developed design from the recurrent neural network (RNN), which can inspect long-term dependencies and has the ability to remember previous information for future usage. The LSTM network has a chain structure consisting of multiple LSTM cells and the proposed DQN structure shown in Figure 2 is clearly adopting the LSTM network as the DNN hidden layers. The DNN based LSTM in Figure 2 is mainly consists of four layers, and each layer contains several neurons, and the weighted sum of each neuron will be the input to an activation function. In our proposed DQN approach, the length of each training sequence is specified as *L*, which is the dimension of the input layer. In our scenario, we choose the input layer of the DNN to include 128 neurons, and the input states to the input layer will be shifted to the subsequent layer after updating the weight parameters [13,22].

As shown in Figure 2, we have applied one LSTM layer as the second layer in both the policy DNN and the target DNN, and the LSTM layer itself includes 300 hidden cells. For each hidden cell, the learnable weights are specified as follows: the input weights *W,* the recurrent weights *R*, and the bias *b*.

The third layer in both the policy DNN and the target DNN is a fully connected layer that processes the outputs of the LSTM layer, and it assembles all of the characteristics and internal information gathered by the prior layers. The fully connected layer behaves separately at each time step, and all neurons in a fully connected layer are connected to all the neurons in the previous layer.

The last adopted layer in both the policy DNN and the target DNN is the regression layer, which is responsible for computing the mean square error (MSE), improving the cell status, and updating the cell weights. A regression layer can also predict the response of the trained network. It is worth mentioning that normalizing the training data in the LSTM network enables the stabilization and acceleration of the training process for neural networks. It is shown in Figure 2, that in the simplified DQN structure, the input states are established according to the size of the input layer, then these states will be passed into both the policy DNN and the target DNN and the state action value functions will be predicted at the output. 

**Figure 3:**
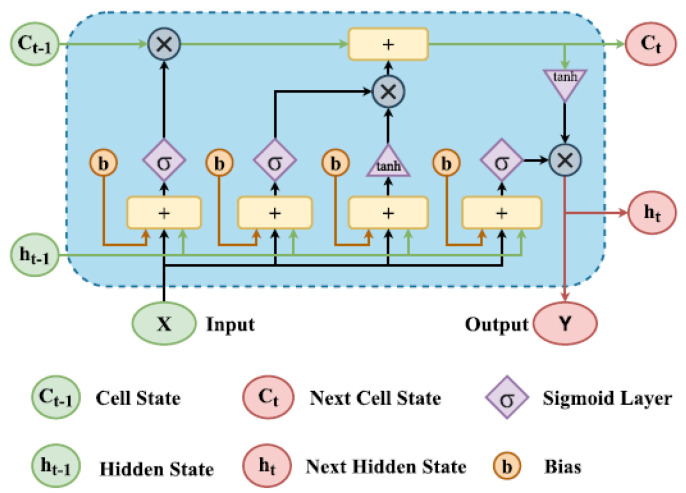
LSTM Cell Structure.

The design of a single LSTM cell is basically shown in Figure 3 [13,22]. Each LSTM cell has three inputs and two output parameters. The hidden state $h_{t-1}$ and the cell state $c_{t-1}$ are the shared parameters between inputs and outputs and the other parameter is the current input. The LSTM cell also includes three sigmoid functions and two tanh functions to regulate the flow of information. In the initialization stage, random hidden states will be generated along with the input for the first LSTM cell. Then the current outputs that include the current hidden state $h_{t}$ and current cell state $c_{t}$ and the new input $x_{t}$ will comprise the three inputs to the next cell.

## 6. DQN Dataset Generation

Typically, the DQN framework involves an agent, a deep neural network (DNN), and the environment. The agent will interact with the environment via the DNN and decide which action to take. In our proposed DQN framework, the BS will be considered as an agent, and it will interact with the environment, which includes the user devices and fading channels. At the start, the agent (BS) will start exploring the environment to collect the information or the states for each user device in the cell, such as power distribution, user distance, channel model, and path loss [23,24].

Typically, at each time step t, and based on the current state $s_{t}$ for each user device, the agent can decide on a certain action $a_{t}$ using the DNN to maximize the sum rates for all users in the NOMA network. Accordingly, the agent (BS) will receive an instant reward $r_{t}$ and move to the next state $s_{t+1}$ in the environment. By taking decisions on actions, the agent (BS) can learn more about the environment to achieve an optimal channel prediction policy $\pi_{c}$. In our scenario, we aim that this optimal policy $\pi_{c}$ for predicting or estimating the channel parameters for each user device can be learned and updated at each time step *t* via the simplified DQN structure illustrated in Figure 2. Furthermore, the agent (BS) can further enhance the policy $\pi_{c}$ by repeating the channel estimation process for multiple episodes. Based on the proposed DQN architecture shown in Figure 2, it is clearly noticed that the DNN based LSTM replaces the Q-table to estimate the Q-values for each state–action pair in the environment, and this designed DNN can be considered as the policy controller for the channel estimation procedure.

## 7. DQN Policy

**Figure 4:**
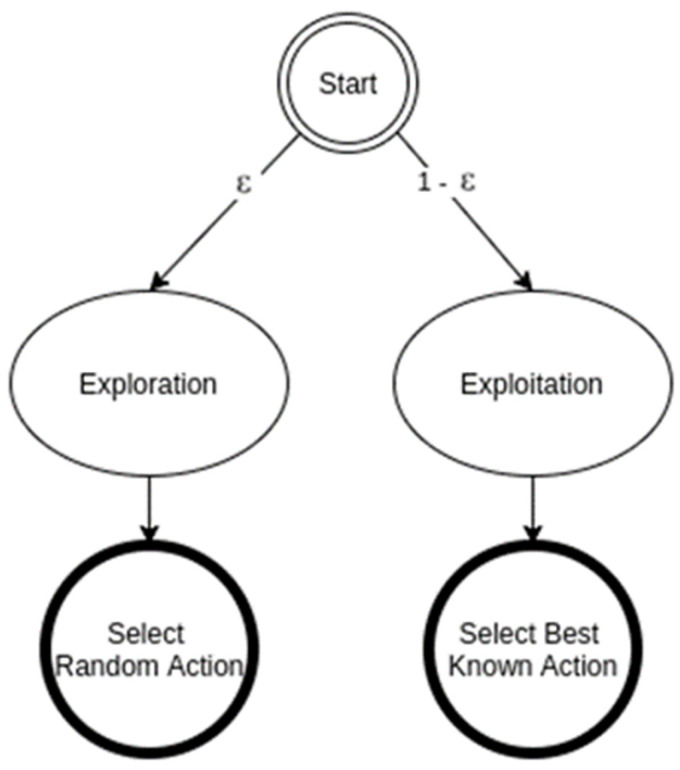
Near-greedy action selection scheme.

The period of time in which the agent interacts with the environment via the proposed DQN scheme is termed an episode, and every episode has a total duration of *T* time steps. At each episode, the main aim is to estimate the channel parameters for each user in order to maximize the sum rates for all users in the NOMA cell. In our simplified DQN approach, the dimension of the input layer for the DNN based LSTM is set equal to the available states in state space ***S*** for each user, and correspondingly the dimension of the output layer is equal to the number of possible actions in the action space ***A*** for each user. As indicated in Figure 2, The LSTM layer, and the fully connected layer are both comprising the hidden layers part of the proposed DQN model, and this may provide a reasonable balance between the network performance and computational complexity. Typically, the Q learning procedure is considered an off-policy algorithm, which means that without applying any greedy policy, the Q algorithm can iteratively estimate the best action for maximizing the future reward. In our developed DQN algorithm, we decide to apply a near-greedy action selection policy, that has two approaches as shown in Figure 4 [25]:

The first approach is the exploration, where the agent discovers and carries out random actions at a time step *t*. The second approach is the exploitation, where the agent can decide on an action to maximize the state-action value function $Q_{\pi }\left(s_{t},a_{t},W_{t}\right)$ based on the previous experience and the current network weights.

In our proposed near-greedy action selection policy, the agent has an exploration rate of ϵ and an exploitation rate of $\left(1-\epsilon \right)$ where 0<ϵ<1, and ϵ is considered a hyperparameter that can control the trade-off between exploitation and exploration during the training process. Hence, based on that designated action selection policy, the agent (BS) can select an explicit action $a_{t}$ at a given state $s_{t}$ at every time step *t* and correspondingly, the agent can receive a positive or negative reward and move to a new state $s_{t+1}$.

## 8. DQN State Space, Action Space, and Reward

Initially, the distance between each user device and the BS and channel path loss needs to be specified in the dataset to facilitate the random generation of the channel coefficients for every user in the examined NOMA system [13,14,22]. In addition, pilot symbols will be created, transmitted, and identified at both the BS and at the receiver side of each device to also assist in the initial channel parameters estimation process. As well, the power factor for each device in the NOMA cell needs to be initially assigned in the dataset. To set up the Q values, the channel parameters for every user device in the cell can be initialized either using the random generation of the channel parameters based on the path loss and the distance or using the pilot symbols. In our simplified DQN algorithm, we initialize the channel parameters based on both schemes, the random generation and pilot symbols. Throughout the DQN algorithm iterations, the Q-values will be predicted according to the DQN algorithm procedure.

As previously mentioned, in our channel estimation procedure, we need to efficiently predict the channel parameters for each user device in the examined NOMA cell to facilitate the maximization of the sum rates for all users in the considered NOMA system at each time step t. Hence, the state space ***S*** can be created to include the following states:

(a) The current power factor $\alpha_{i}$ for each user in the NOMA cell,
(b) The current user distance $d_{i}$ that represents the distance between BS (agent) and the user device i.
(c) The present channel path loss φ.

Accordingly, the resultant state space ***S*** for *N* users NOMA system can be represented as [13,14,25]

(22) $$S=\left\{\alpha_{1}\;\alpha_{2}\;\alpha_{3}\;\ldots \alpha_{N}\;d_{1}\;d_{2}\;d_{3}\ldots d_{N\;},\;\varphi \;\right\}\;$$

For each user, all the actions that can be chosen by the agent (BS) can be selected from action space ***A***. In our scanario, the possible actions in the action space ***A*** can be introduced as follows:

(a) Change the distance of the user device within a limited range of 5 m.
(b) Increase or decrease the power distribution factor $\alpha_{i}$ by a certain step size of 0.05.

The reward function also plays an principal role in the DQN algorithm, and there are many ways to assign the rewards based on the selected action. In our developed DQN scenario, we decided to calculate the rate for each user in the NOMA system using (1), to reflect the immediate reward r returned from the environment to the agent (BS) after choosing a certain action $a_{t}$ at state $s_{t}$. Hence, based on the selected action, if the calculated data rate is higher than a specified threshold $R_{th}$, this will reflect a positive reward for the agent, while a lower data rate will reflect a negative reward. Based on the aforementioned discussion, Algorithm 1 can summarize the algorithm steps for estimating the channel parameters for each user in the NOMA cell, based on our simplified DQN structure.
**Algorithm 1 Proposed DQN Algorithm for channel parameters estimation**Initialize policy DNN and target DNN networks with random weights $(W,\;\hat{W)}$.Initialize experience replay memory (ERM).Randomly generate the exploration rate ϵ.**for** each episode do    **for** each step do       **for** each user device do           based on ϵ, and based on the current state $s_{i}$,            Select the channel parameters and add to action space $a_{i}$
       **end** forObserve the immediate rewards $r_{i}$ and move to the next state $s_{t+1}$.Insert $\left(s_{i},a_{i},r_{i},s_{t+1}\right)$ in experience replay memory (ERM).Create a mini batch with random sample of tuple $\left(s_{i},a_{i},r_{i},s_{t+1}\right)$ from ERM.**for** each tuple in mini batch do    Predict the Q-values using policy DNN.    Approximate $Q^{*}$ values using target DNN.    Calculate the loss between $Q^{*}$ values generated from Target DNN and *Q*
     values generated from Policy DNN.     Update the weights W of the policy DNN using SGD.**end** for**end** for$\hat{W}\leftarrow \;W$ after a certain number of *T* steps.**end** for

## 9. Detailed DQN Procedure and Workflow

In this section, we can list the detailed workflow for the developed DQN algorithm that is responsible for estimating the channel parameters for each user in the examined NOMA system:

- Initialize the weights for both the policy DNN and the target DNN.
- Initialize the ERM with a typical size of 10,000 (it can be 10^6^).
- Initialize the ϵ parameter for near-greedy action selection policy with a large value of ϵ=0.999  (start by exploration then decay).
- Initialize data records (tuples).
  (a) Generate a random channel coefficients based on the fading model parameters with size = 120).
  (b) Based on the pilot symbols, approximate the channel coefficients with size = 8).
  (c) For each user, both the randomly generated channel parameters and the coefficients generated based on the pilot symbols will be combined and used as initial channel parameters.
- Assign initial distance, initial power factor, and path loss, and prepare the state space S for each user.
- Select a random state $s_{t}$ from the sate space and used it as an input for policy DNN.
- The policy DNN will select a random action and correpondigly select a random Q-value, and based on this step, the policy DNN can predict the channel coefficients.
- Calculate the rate, and based on the calculated rate the reward can be assigned.
- Go to the next state $s_{t+1}$
- Compose a tuple $e_{1}=\left(s_{t},\;a_{t},\;r,\;s_{t+1}\right)$
- Store a tuple $e_{1}$ in ERM.
- Generate experience tuples = 1000, and store these tuples in ERM.
- Select a random batch of the tuples from ERM with batch size 32 tuples.
- Number of episodes = 20, and number of steps = 10^4^
- For each tuple in the random batch do the following:
  (a) From the policy DNN, select the Q-values (channel coefficients) randomly.
  (b) From the Target DNN select the Q-values based on the greedy policy
  (c) Assign the Reward.
  (d) Calculate the Loss function as follows:
    (Target Q-value (Reward + Q_max_ value) − policy Q-value).
  (e) Update the weights of the policy DNN based on the SGD.
- Every *T* = 10^2^ steps, copy the weights of the policy DNN to the Target DNN.
- Activation functions used in LSTM layers are (sigmoid and tanh), while activation functions used at the output layer are (linear or Relu).
- SGD optimizer is utilized for weight updates.

## 10. Complexity Analysis

It is important to quantify the computational complexity of the proposed algorithm. Overall, deep learning algorithms are mainly dependent on hyperparameters, hence, applying analytical methods to guarantee the convergence of the proposed DQN algorithm usually has some sort of difficulty. Hence, it is a common challenge in literature to prove the optimality and convergence of the algorithm in an analytical way [26,27,28]. Alternatively, in this section we can focus on showing the amount of work per iteration in the developed DQN algorithm. For the NOMA system with *N* users and *K* base stations, the computational complexity of the proposed DQN algorithm can be introduced as follows: it is known that the size of the state space is denoted by S and the size of the action space is denoted by A and both have a significant role in the complexity of the deep Q-learning algorithm. Following [14,29], the computational complexity of the Q-learning algorithm with the greedy policy is estimated to be $O\left(S\times A\times M\right)$ for each iteration, where S is the number of states, A is the number of actions, and M is the number of steps per episode. In our proposed DQN scenario, it can be shown that the size of the state space is *K* + *N*, and the size of the action space is 2(*K* + *N)*. Therefore, the amount of the work per iteration can be described as follows $O\left(\left(2K^{2}+\times 4NK+2N^{2}\right)\times M\right)$. According to [12], the corresponding computational complexity for the traditional channel estimation method based on MMSE procedure can achieve a relatively low complexity $O\left(N^{2.37}\right)$ [12,30] but at the cost of performance degradation. Based on the aforementioned analysis, it can be shown that the developed DQN algorithm has some sort of complexity but at the cost of performance improvement as will be verified in the simulations results.

## 11. Simulation Parameters and Environment

Discussion for the simulation parameters and settings is described in this section. The simulated downlink NOMA system includes three distinct user devices and one BS. The BS is equipped with a single antenna and each user device in the cell is also equipped with a single antenna. In the simulated NOMA environment, the modulated signal related to each user in the downlink transmission will be superimposed and transmitted by the BS to each user device via independent Rayleigh fading channels, and the path loss is set to 3.5. At the receiver side, we assume that a perfect SIC procedure is applied and AWGN is considered and the noise power density is set to $N_{0}=-174$ dBm/Hz.

MATLAB simulation tool is employed to realize the following: (1) inspect, characterize, and evaluate the performance of the proposed deep reinforcement learning based DQN algorithm which developed to be utilized as a channel estimator in the examined NOMA system, (2) Diverse performance metrics will be measured to evaluate the efficiency of the proposed DQN algorithm when being utilized in the channel estimation process. Simulations are accomplished with $10^{4}$ iterations, and limited pilot symbols are generated and recognized at the BS and each user device to assist in the estimation process. The main simulation parameters can be summarized as shown in Table 1.

The simulation figures are created based on the assumption that the channel parameters for each user will be estimated based on the simplified DQN algorithm. Therefore, in order to examine the impact of utilizing the proposed DQN approach, the channel estimation technique based on standard minimum mean square error (MMSE) procedure [12] is also simulated for the sake of comparison. As indicated in Section 9, initially both the randomly generated channel parameters and the channel coefficients generated based on the pilot symbols will be combined and used in the simulation environment, to model the Rayleigh fading channel. In our developed DQN algorithm, at the end of each training episode, the predicted $Q\left(s,a\right)$ values generated from the policy DNN will be employed as an approximated channel coefficients for each user device to recover the desired signal. Different power factors are initially assigned for every user device according to the current distance from the BS and the present channel condition. Power factors $\alpha_{n}$, $\alpha_{m}$, and $\alpha_{f}$ are assigned for near, middle, and far users, respectively. In a fixed power allocation setup, we initially assign $\alpha_{f}=0.65$, $\alpha_{m}=0.3$, and $\alpha_{n}=0.05$. In the simulation files, the transmission distance for every user device with respect to BS is initially defined as follows: $d_{f}=1000\;m$, $d_{m}=500$ m, and $d_{n}=100$ m. User data and pilot symbols are modulated using the Quadrature phase shift keying (QPSK) modulation format and the applied transmitted power range is set to vary from 0 to 30 dBm for many reasons, firstly, to match with the benchmark environments that simulated from the literature, secondly, most of the simulation environments are applying this classical range, and thirdly, on average, the performance metric behavior can be certainly predictable after 30 dBm power level.

## 12. Simulation Results and Analysis

**Figure 5:**
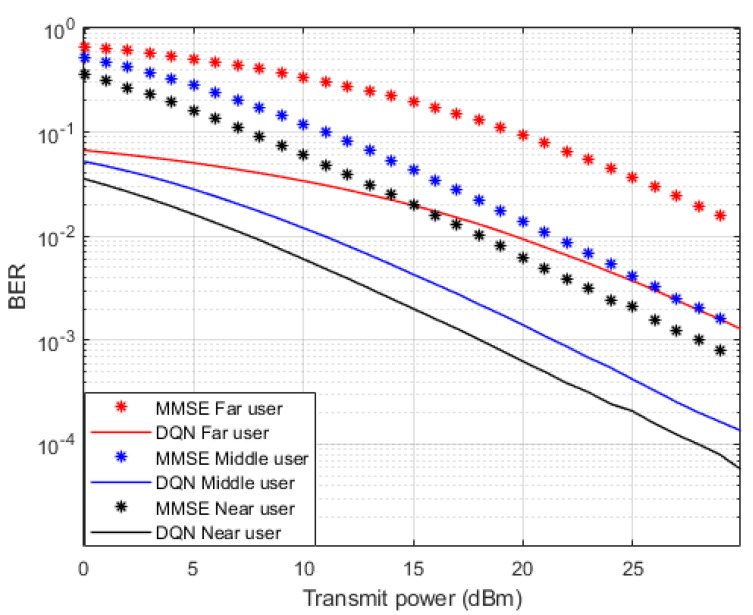
BER vs. power (DQN—MMSE).

Simulation results that describe the comparison between the proposed DRL based DQN algorithm and the MMSE procedure when both being utilized to estimate the channel parameters for each device are shown in Figure 5 in terms of BER versus power. The estimated channel parameters using both procedures will be employed for the signal recovery for each user and the simulated results are generated where a fixed power allocation scheme is considered. It is clearly noticed that when the developed DQN algorithm is applied for predicting the channel parameters, each user device in the examined NOMA cell shows the ability to provide a visible enhancement in lowering the BER compared to the MMSE technique. As an example, at a particular transmitted power of 20 dBm, the realized BER value for far user device using the MMSE procedure is 10^−1^, while the achieved BER in the case of DQN is 10^−2^. Similarly, the improvment in the BER for middle and near user devices is obviously observed when the simplified DQN algorithm is applied compared to the MMSE procedure.

**Figure 6:**
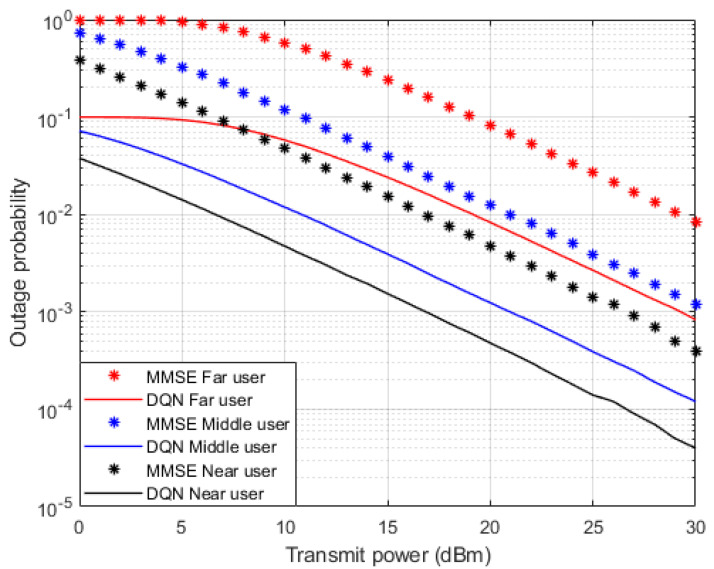
Outage Probability vs. power (DQN—MMSE).

In terms of the outage probability against applied power, Figure 6 illustrates the simulation results for the inspected user devices in NOMA cell when both the simplified DQN algorithm and the standard MMSE technique are implemented separately as a channel estimators. Similar to BER results, all user devices simulation outcomes indicate about 10 dBm enhancement in the power saving when the proposed DQN algorithm is applied compared to the MMSE technique. The reduction in the power transmitted also supports the improvement achived in minimizing the outage probability when the DQN algorithm is adopted. These visible improvements verify the advantage of usage the simplified DQN scheme as a channel estimator compared to the traditional MMSE procedure.

**Figure 7:**
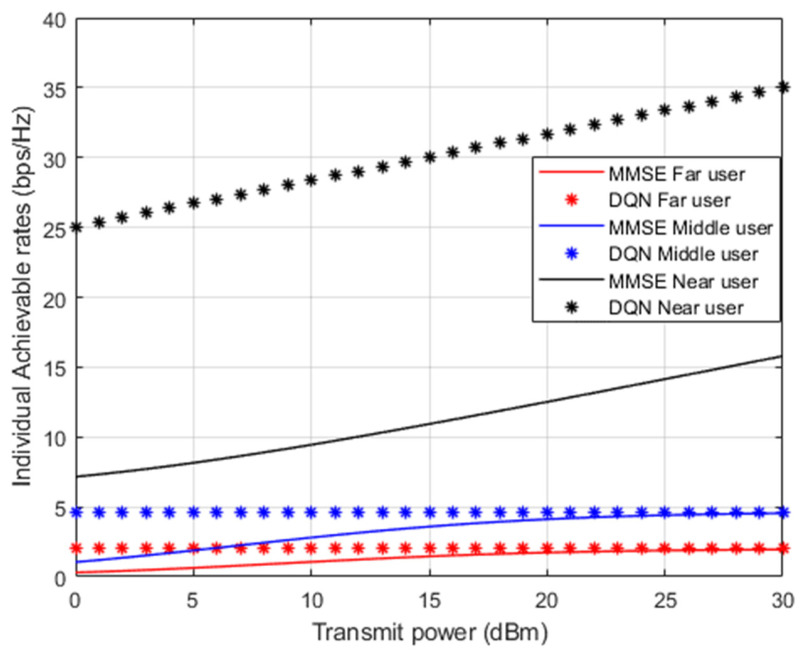
Achievable rates vs. power (DQN—MMSE).

Figure 7 presents the simulation results for the attainable capacity for each user in the examined NOMA system when both the simplified DQN algorithm and the standard MMSE channel estimation procedures are applied separately. The achieved rate for the near device shows significant enhancement by about 20 bit/s/Hz compared to far and middle users’ rates. The dominance of the near user in terms of the possible rate may be justified by the stable channel condition for the near user compared to other users in NOMA system. Moreover, the results indicate that the proposed DQN algorithm still can deliver a stable bit rate compared to the MMSE technique for far and middle users’ scenarios, and this slight improvment can be justified by the interference factor and inadequate link conditions for far and middle users.

**Figure 8:**
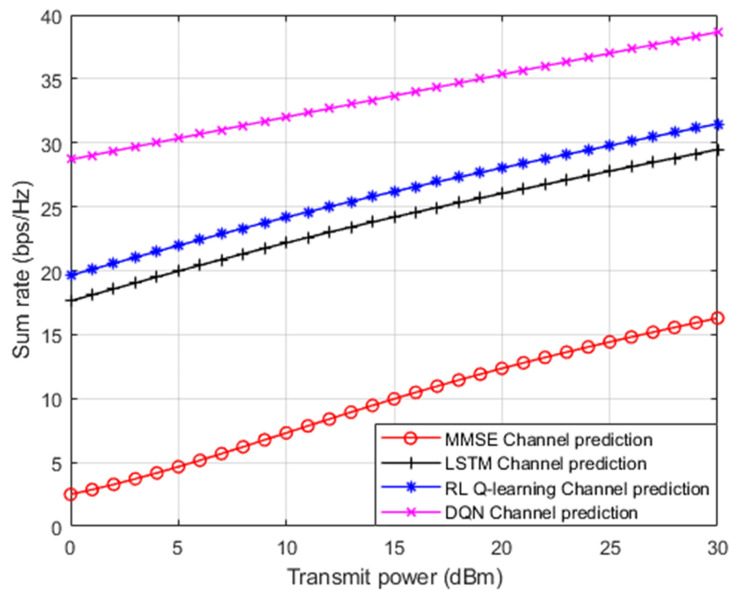
Sum rate vs. power (MMSE, LSTM, RL Q-learning, DQN).

In Figure 8, three distinct channel prediction schemes are investigated here as a benchmark comparison: (1) standard minimum mean square error (MMSE) procedure for channel estimation [12]; (2) DL based LSTM network for channel prediction applied in [13]; and RL based Q algorithm for channel estimation applied in [14]. Figure 8 displays the simulation outcomes for the sum rate for all user devices in the examined NOMA cell versus the applied power. It is apparent that the developed DRL based DQN algorithm shows superiority over the standard MMSE procedure approximately by more than 20 bit/s/Hz. Furthermore, the simplified DQN algorithm shows an improvement over the DL based LSTM procedure presented in [13] by nearly 10 bit/s/Hz. For the third benchmark applied in [14], the simplified DQN procedure shows a performance enhancement by 8 bit/s/Hz, approximately compared to the RL based Q algorithm. These findings support that this simplified DQN algorithm can be a strong candidate technique compared to other procedures when it is being utilized as a channel estimator.

**Figure 9:**
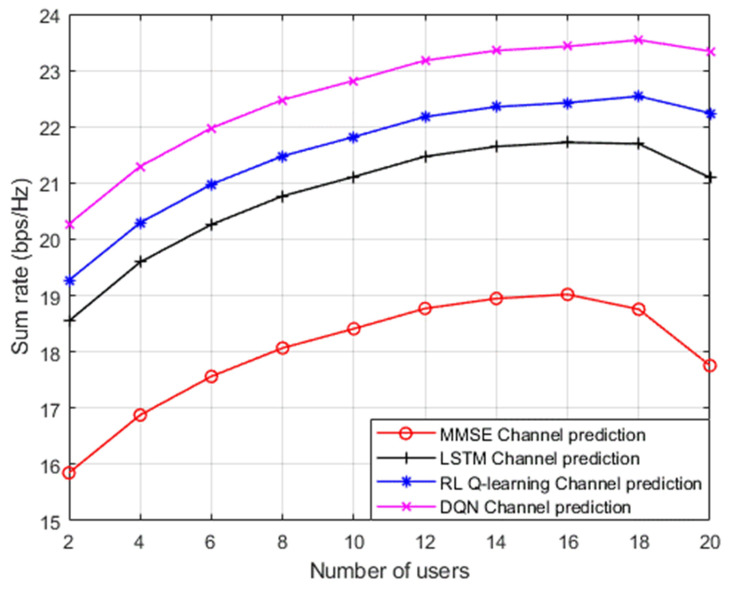
Sum rate v. number of users (MMSE, LSTM, RL Q-learning, DQN).

Simulation results for the sum rate performance metric against different numbers of users in the examined NOMA cell are also illustrated in Figure 9, where the reference power is assigned to be 1 dBm. Similar to the simulation environment in Figure 8, three distinct channel prediction schemes are also investigated here as a benchmark comparison: (1) channel estimation based on standard minimum mean square error (MMSE) procedure [12]; (2) DL based LSTM structure for channel prediction applied in [13]; and RL based Q algorithm for channel estimation applied in [14]. As revealed from the results in Figure 9, it is clearly noticed that our simplified DQN algorithm can realize a substantially greater sum rate with respect to the MMSE procedure, by at least 4 bit/s/Hz when the cell capacity is initialized with 2 users. It can also be noticed that as the number of user devices in the cell keeps increasing, the developed DQN algorithm still shows dominance in accomplishing higher sum rates compared to the DL based LSTM scheme by 2 bit/s/Hz approximately. Similarly, the hidden layers feature in the simplified DQN scheme play a sufficient role in providing a noticeable enhancement in the sum rates compared to the Q-learning algorithm while the number of user devices in the NOMA cell is increasing. Generally, these findings reveal that dependability can be ensured by our simplified DQN algorithm even when the user devices in the cell increase. Furthermore, it is worth saying that while increasing the user devices in the cell, the interference will also grow up, thus the performance and the sum rate could be affected.

**Figure 10:**
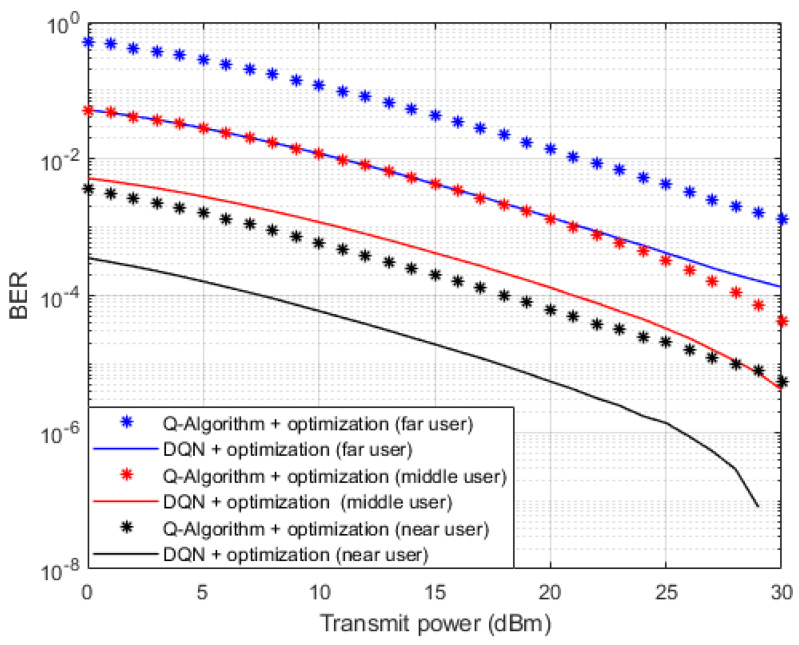
BER vs. power (DQN—Q learning—Optimization).

Simulation results in terms of BER against the applied power are also shown in Figure 10, where both the proposed DQN approach and the RL based Q algorithm [14] are utilized as different approaches for channel parameters estimation. Moreover, the optimized power coefficients derived in [13] for the examined NOMA cell are also applied in this simulation environment. Simulation outcomes indicate that all user devices in the cell can provide a perceivable enhancement in the performance when the simplified DQN algorithm is applied as a channel estimator compared to the case when the Q learning algorithm is implemented when the optimized power scheme is considered. Based on the simulation results, it can be clearly noticed that the developed DQN algorithm for channel estimation and the optimized power scheme can both provide an imprvment in the power saving by more than 5 dBm compared to the case when Rl based Q algorithm and optimized power scheme are both applied.

## 13. Conclusions

In this paper, the impact of utilizing a simplified deep reinforcement learning based DQN algorithm to specifically estimate the channel parameters for each user device in the NOMA system is discussed. In the proposed algorithm, the DQN model is initialized based on generating a random channel parameters then the weights of the simplified DQN model are updated based on the interaction between the agent and the environment in order to maximize the received downlink sum rates and at the same time minimize the loss function. The reliability of the developed DQN structure to estimate the channel parameters is examined by comparing the performance of the proposed DQN algorithm with a diverse benchmark schemes. A selective benchmark schemes were simulated, such as MMSE procedure for channel estimation, DNN based LSTM for channel estimation, and RL based Q algorithm for channel estimation. Simulation outcomes have proven that the simplified DQN algorithm can provide a noticeable enhancement in terms of the system performance compared to the simulated benchmark schemes. Furthermore, various performance metrics have been examined, and the simulation results also verified the superiority of the simplified DQN structure even when the cell capacity is increased.

## Figures and Tables

**Table 1:** Summary of Simulation Parameters.

Parameter	Value
Simulation Tool	MATLAB
Modulation type	QPSK
Number of Users	3, [2–20]
System Bandwidth *B*	1000 kHz
Fading distribution	Rayleigh
Path loss φ	3.5
Number of Iterations	10^4^
Noise PSD $N_{0}$	−174 dBm/Hz
Learning Rate α	0.01
Discount factor γ	0.9
Batch size	32
Initial exploration rate ϵ	0.999
Optimizer	SGD
$R_{th}$	2 b/s

## Data Availability

Not applicable.

## Abbreviations

The following abbreviations are used in this manuscript:

AWGN	Additive White Gaussian Noise
BER	bit error rate
BS	Base Station
CSI	Channel state information
DL	Deep Learning
DNN	Deep Neural Network
FPA	Fixed Power Allocation
OPS	Optimized Power structure
LSTM	Long Short-Term Memory
DQN	Deep Q networks
ML	Machine Learning
MSE	Mean Square Error
MMSE	Minimum Mean Square Error
MUD	Multiuser detection
PD-NOMA	Power Domain Non-Orthogonal Multiple Access
QoS	Quality of Service
SIC	Successive interference cancellation
RL	Reinforcement Learning
DRL	Deep Reinforcement Learning
